# 4-1BB Agonists: Multi-Potent Potentiators of Tumor Immunity

**DOI:** 10.3389/fonc.2015.00117

**Published:** 2015-06-08

**Authors:** Todd Bartkowiak, Michael A. Curran

**Affiliations:** ^1^Department of Immunology, University of Texas MD Anderson Cancer Center, Houston, TX, USA; ^2^The University of Texas Graduate School of Biomedical Sciences at Houston, Houston, TX, USA

**Keywords:** 4-1BB, immunotherapy, CD137, co-stimulation, combination therapy

## Abstract

Immunotherapy is a rapidly expanding field of oncology aimed at targeting, not the tumor itself, but the immune system combating the cancerous lesion. Of the many approaches currently under study to boost anti-tumor immune responses; modulation of immune co-receptors on lymphocytes in the tumor microenvironment has thus far proven to be the most effective. Antibody blockade of the T cell co-inhibitory receptor cytotoxic T lymphocyte antigen-4 (CTLA-4) has become the first FDA approved immune checkpoint blockade; however, tumor infiltrating lymphocytes express a diverse array of additional stimulatory and inhibitory co-receptors, which can be targeted to boost tumor immunity. Among these, the co-stimulatory receptor 4-1BB (CD137/TNFSF9) possesses an unequaled capacity for both activation and pro-inflammatory polarization of anti-tumor lymphocytes. While functional studies of 4-1BB have focused on its prominent role in augmenting cytotoxic CD8 T cells, 4-1BB can also modulate the activity of CD4 T cells, B cells, natural killer cells, monocytes, macrophages, and dendritic cells. 4-1BB’s expression on both T cells and antigen presenting cells, coupled with its capacity to promote survival, expansion, and enhanced effector function of activated T cells, has made it an alluring target for tumor immunotherapy. In contrast to immune checkpoint blocking antibodies, 4-1BB agonists can both potentiate anti-tumor and anti-viral immunity, while at the same time ameliorating autoimmune disease. Despite this, 4-1BB agonists can trigger high grade liver inflammation which has slowed their clinical development. In this review, we discuss how the underlying immunobiology of 4-1BB activation suggests the potential for therapeutically synergistic combination strategies in which immune adverse events can be minimized.

## Introduction

Current front-line therapies in the treatment of cancer seek to destroy large tumor lesions by either inducing irreparable DNA damage in the case of radiation therapy and certain chemotherapeutic agents (e.g., alkylating agents, anthracyclines), or by inhibiting protein synthesis, transport, or cell cycle progression (e.g., antimetabolites, topoisomerase inhibitors, mitotic inhibitors, proteasome inhibitors). Radiotherapy has proven effective against a variety of hematological and epithelial cancers including leukemia and lymphoma, head and neck, breast, cervical and prostate cancer. Similarly, DNA alkylating agents and antimetabolites are being used to treat lymphomas, leukemias, brain cancers, and some carcinomas. In most cases, however, tumors become refractory to treatment, leading to the development of therapy-resistant metastases. Furthermore, the delicate radio-sensitivity of many organs, inaccessibility of lesions to surgical resection, and toxicity of chemo- and radio-therapeutic agents further complicate the use of such therapies.

The ground-breaking revelation that the body’s own immune system can recognize tumor antigens as foreign and initiate anti-tumor responses against growing lesions has changed the field of tumor therapy (1, 2); boosting the immune response against tumors has become an exciting new avenue in the fight against cancer. Immunotherapy of cancer, as opposed to drug therapy, provides the added benefit of sustained protection often with less severe and less persistent side effects compared to those associated with chemotherapeutics or radiation therapy. Early immunotherapies sought to boost systemic immune responses by administration of prosurvival or proinflammatory cytokines with varied, and often suboptimal clinical outcomes (3–6). These early therapies failed, in part, due to our inadequate understanding of the immune relevance of conserved versus mutanome antigens, tumor immune escape mechanisms, and, foremost, the complex and highly immunosuppressive tumor microenvironment. With a better understanding of how the immune system targets tumors and tumor antigens, an entire arsenal of immunotherapeutics has been developed to augment anti-tumor immune responses from vaccine strategies to adoptive transfer of tumor-reactive T cells. The most successful approaches that have been translated from bench to bedside, however, aim at targeting co-receptors expressed on various immune cells in the tumor microenvironment. Therapeutic antibodies which block the co-inhibitory checkpoint receptors on T cells were among the first, and most effective, of the current generation of therapeutics targeting the immune system. Ipilimumab (αCTLA-4) became the first FDA approved T cell checkpoint antibody for use against melanoma, with patients showing a 13% objective response rate against Stage III/IV melanoma, with largely manageable immune-related adverse events (7–9). The approval of Ipilimumab paved the way for the transition of other checkpoint blockade antibodies into clinical trials. Nivolumab and Pembrolizumab [both antagonist antibodies targeting the programmed death receptor-1 (PD-1)] are currently approved for melanoma, and, more recently, non-small cell lung cancer (NSCLC) in the case of Nivolumab. Other trials of PD-1 blockade are ongoing for renal cell carcinoma (RCC), NSCLC (NCT01844505, NCT02041533, NCT01866319, NCT02212730), and glioblastoma (NCT02311920, NCT02311582) among others. Tumor-infiltrating lymphocytes (TIL) express a wide array of additional co-stimulatory and co-inhibitory receptors, though, that may serve as potential targets for novel immunotherapeutic interventions (10). One such immuno-stimulatory receptor with promising clinical applications is the tumor necrosis factor superfamily member 4-1BB (CD137/TNFRSF9).

Targeting 4-1BB with agonist antibodies elicits potent anti-tumor responses; however, clinical progress has been slowed by dose-limiting liver inflammation. This review will explore the current knowledge of the function of 4-1BB and its role in the immune response, potentiating both antiviral and antitumor responses, while alleviating certain autoimmune conditions. The means by which the 4-1BB co-receptor can be targeted to induce anti-tumor immunity will be highlighted, with a particular focus on the unique potential for synergism between 4-1BB co-stimulation and various other immune and non-immune therapies.

## The Role of 4-1BB in the Immune Response

**Figure 1:**
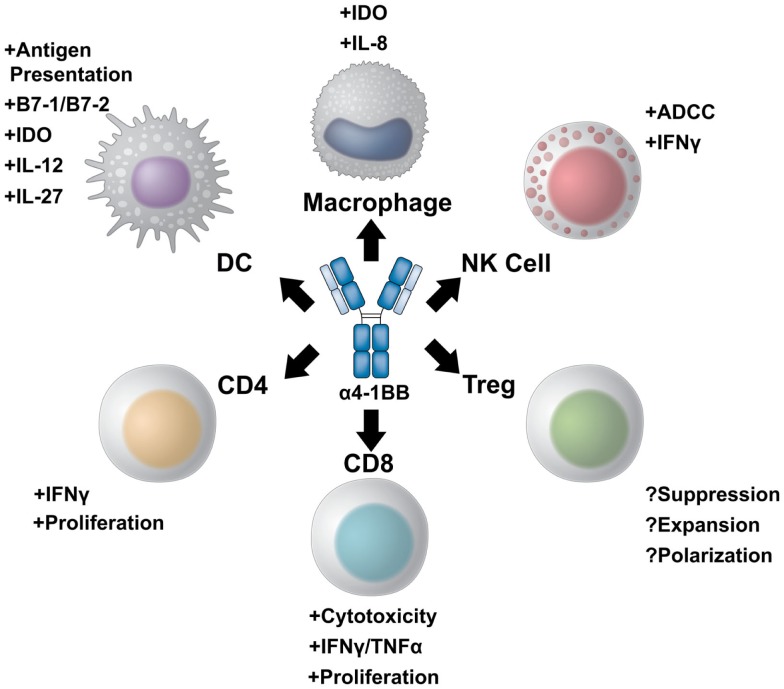
**A multi-potent role for 4-1BB targeted immunotherapy**. 4-1BB agonist therapies elicit diverse immune effector responses on both the innate and adaptive immune arms. The most potent of responses stimulate CD8^+^ cytotoxic T cells to proliferate and increase their effector potential through increased interferon gamma production and expression of multiple granzymes. CD4^+^ effector T cells can also be stimulated to expand and produce pro-inflammatory cytokines. The role of 4-1BB stimulation on regulatory T cells, however, is controversial. 4-1BB agonist therapy may either inhibit differentiation of conventional effector cells into Tregs while also inhibiting Treg suppression, or, conversely, maintain Treg expansion and suppressive capacity. NK cells also benefit from 4-1BB agonist therapy. Not only can α4-1BB antibodies stimulate antibody-dependent cell-mediated cytotoxicity through Fc/FcR interactions, but activated NK cells express 4-1BB to become targets of therapy. Additionally, cells of the myeloid lineage upregulate 4-1BB upon activation. 4-1BB agonists targeting dendritic cells induce DC maturation and antigen presentation. In addition, α4-1BB stimulated DCs begin to express IL-12 and IL-27 as well as the enzyme IDO to modulate T cell function. 4-1BB^+^ macrophages can also be stimulated to increase antigen presentation and produce IL-8 as well as IDO.

4-1BB belongs to the TNF receptor family, which includes multiple T cell co-stimulatory receptors which have been targeted with agonist antibodies including GITR, CD40, CD27, HVEM, LIGHT, APRIL, and TWEAK (11, 12). 4-1BB plays a critical role in sustaining effective T cell immune responses and in generating immunological memory. The expression profile of 4-1BB, as well as its unique ability to potentiate robust effector responses in multiple subsets of lymphocytes relevant for tumor immunity, makes 4-1BB a uniquely appealing target for immunotherapy (Figure 1).

### Expression patterns of 4-1BB

4-1BB is expressed on a multitude of cells of the hematopoietic lineage (13). While 4-1BB is widely known to be transiently upregulated on CD8 T cells following activation (14–18), 4-1BB can also be expressed on activated CD4 helper T cells (17, 19, 20), B cells (21, 22), regulatory T cells (23), natural killer (NK) cells (24–26), natural killer T (NKT) cells (27), gamma-delta T cells (28), dendritic cells (29, 30), mast cells (31), osteoclasts (32, 33), thymocytes (34), early myeloid progenitor cells (35, 36), and activated endothelium (37–40). Human neural cells also express 4-1BB, including neurons, astroglia, and microglia in the brain (41). Multiple subtypes of lymphomas and leukemias are also known to express 4-1BB, although its precise function remains unclear in the setting of malignancy (42–45). The broad range of 4-1BB expression on multiple cell types makes this receptor a dual-edged sword in the fight against cancer; as stimulation with 4-1BB agonists elicits strong anti-tumor responses from a myriad of cell types, however, sometimes at the cost of off-target immune pathology. Significant progress has been made in recent years in describing the complex regulation of 4-1BB expression; yet, further studies are needed to clarify the impact of each 4-1BB expressing cell population toward the anti-tumor versus auto-inflammatory effects of 4-1BB agonist antibodies.

### 4-1BB signaling

Co-stimulation through the 4-1BB receptor activates multiple signaling cascades within the T cell, powerfully augmenting T cell activation. Upon receptor ligation, 4-1BB enhances signaling through the T cell receptor (18). 4-1BB forms a heterotrimer complex consisting of two TNF-receptor associated factor (TRAF)-2 complexes (46) in conjunction with TRAF-1 (47). This interaction, through leukocyte specific protein-1 (LSP-1) (48), potentiates signaling through the c-Jun N-terminal kinase (JNK) pathway (49), the extracellular signal-regulated kinase (ERK) pathways (50, 51), as well as through β-catenin and AKT (51). These signaling pathways converge on the master transcription factor NF-κB to regulate 4-1BB signaling, as well as effector immune responses (47, 52). As most of these signals are shared between TNF co-stimulatory receptors, yet none of the others can replicate the phenotypic changes associated with 4-1BB activation, it is likely that additional molecular pathways are triggered by 4-1BB which have yet to be described.

### Potentiation of effector immune responses

4-1BB signaling inhibits activation-induced cell death (AICD) in T cells (53), promotes survival (20), is critical for the formation of immunological memory through upregulation of the anti-apoptotic genes Bcl-2, Bcl-xl, and Bfl-1 (16, 17), and induces T cell proliferation and enhanced effector function (14). Interestingly, T cells from 4-1BB deficient mice demonstrate enhanced proliferative potential, while exhibiting reduced effector responses (54, 55). Moreover, 4-1BB^−/−^ mice demonstrate defects in myelopoiesis as well as B cell deficiencies in the production of IgG2a and IgG3 (55).

In addition to enhancing IFNγ and TNFα production (56, 57), activation of 4-1BB has been shown to induce IL-13 production from both CD8 and CD4 T cells to limit inflammation (58). Moreover, enhanced IFNγ production leads to the generation of indoleamine 2,3-dioxygenase (IDO) by dendritic cells which can attenuate 4-1BB-mediated effector responses (59). In addition, 4-1BB signaling induces maturation of dendritic cells leading to the upregulation of B7 co-stimulatory ligands, increases DC survival, and boosts the production of inflammatory cytokines such as IL-6, IL-12, and IL-27 (60, 61). Further, DCs present in the mesenteric lymph nodes upregulate retinal dehydrogenase through 4-1BB signaling, which promotes Treg development in order to maintain homeostasis in the gut (62).

## A Dual Role of 4-1BB in Infectious Disease and Autoimmunity

The ability of 4-1BB receptor signaling to evoke robust effector responses has been extensively demonstrated in infectious disease models. Several studies have shown a role for 4-1BB in mediating antiviral immune responses toward influenza (63–65), hepatitis C (66), cytomegalovirus (67), HIV (68), lymphocytic choriomeningitis virus (69), as well as poxviruses (70, 71). Additionally, 4-1BB mediates anti-bacterial immune responses particularly toward *Streptococcus pneumoniae* (72) and *Listeria monocytogenes* infection (73, 74).

Although 4-1BB potentiates strong immune responses, it also has the potential to alleviate autoimmune disease. Stimulation through 4-1BB ameliorates murine models of experimental autoimmune encephalomyelitis (EAE) (75, 76), systemic lupus erythematosus (SLE) (77–79), murine Sjögren’s disease (80), inflammatory bowel disease (81, 82), uveoretinitis (83), and rheumatoid arthritis (84). Conversely, 4-1BB may worsen type I diabetes (85–87), although one study demonstrated a role for 4-1BB in protecting mice from pathology by increasing CD4^+^CD25^+^ regulatory T cells (88). Further, 4-1BB may also play a role in alleviating allergic reactions (89, 90). The capacity of 4-1BB to mediate both potent immune responses and ameliorate autoimmunity likely stems from the unique ability this receptor possesses to promote Th1 type responses, while inhibiting Th2- and Th17-related pathologies (61, 76).

## Targeting 4-1BB in Immuno-Oncology

The dual ability of 4-1BB to stimulate strong effector T cell responses toward pathogens while restricting autoimmune disease has made this receptor an attractive target for cancer immunotherapy. While 4-1BB monotherapy is capable of mediating significant tumor regressions and even cures of numerous types of established murine tumors (Table 1), targeting 4-1BB with agonist antibodies as a monotherapy in the clinic has only yielded modest frequencies of RECIST partial responses and stabilization of disease. Although agonist antibodies have been the best studied modality for activating 4-1BB, the immune pathologies associated with their use have prompted the development of alternate therapeutics seeking to separate 4-1BB’s anti-tumor effects from its associated liver inflammation (91). Each of these potential drugs for activation of 4-1BB has unique advantages and disadvantages for use in combination with other therapies.

**Table 1:** **Combinations with 4-1BB targeted therapies**.

Combination	Tumor Model	Result	Reference
*Viral Gene Therapy*			
pAd/RSV-mIL-12	Metastatic MCA26 colon carcinoma	-cured hepatic and lung metastases -NK & CD8^+^ mediated	(104)
Adv./IL-12 +Adv./4-1BBL	Metastatic MCA26 colon carcinoma	-**↑** survival -NK &CD8^+^ mediated	(105)
	Metastatic JC breast carcinoma	-**↑** survival -Critical role for CD4^+^ Th	(131)
pLXSHD.m4-1BBL	AG104A sarcoma P815 mastocytoma	-**↑** CTL activity -complete rejection with CD28 stimulation	(130)
AdCMVmIL-12 DCs + α4-1BB mAb	CT26 colon adenocarcinoma MC38 colon adenocarcinoma	-complete rejection -systemic immunity	(150)
Adv/IL-12	B16-F10 melanoma EL4 lymphoma	-**↑** CTL activity -NK & CD8^+^ mediated -↓ pulmonary metastases	(106)
Adv-mIL-12 + Adv/Ig-4-1BBL	MCA26 colon carcinoma	-complete regression -long-term survival	(182)
Ad-δB7/IL-12/4-1BBL	B16-F10 melanoma	-↓ tumor burden -**↑** Th1 responses	(92)
Vaccinia virus + α4-1BB mAb	AT-3 breast carcinoma MC38 colon carcinoma	-↓ tumor burden -**↑** myeloid infiltrate in tdLN -**↑** CD8^+^ T cell, NK & neutrophil tumor infiltrate	(93)
rV-4-1BBL + lymphodepletion	B16-F10 melanoma	-**↑** MHC-I expression -↓ antiviral antibodies -**↑** viral persistence -**↑** CD8^+^ effector memory	(103)
rV-4-1BBL	MC38-CEA^+^ colon carcinoma	-↓ tumor burden -**↑** Th and CTL responses	(100)
Semliki Forest Virus-IL-12 + 4-1BB mAb	B16 melanoma TC-1 HPV^+^ lung adenocarcinoma	-75% complete regression -**↑** tumor-specific CTL -↓ anti-vector humoral response	(98)
*Checkpoint Blockade*			
αCTLA-4+ α4-1BB	B16 melanoma	-**↑** survival with FVAX -**↑** CD8^+^ T cell infiltrate -↓ Treg infiltrate -**↑** T cell function -**↑** T cell/MDSC ratio	(183)
	B16 melanoma MC38 colon carcinoma	-ineffective against melanoma -CD8^+^ T cell mediated rejection of colon carcinoma -↓ autoimmune side effects -**↑** Treg activity	(110)
αCTLA-4 + 4-1BBL-tumor vaccine	RM-1 prostate carcinoma	-**↑** survival -complete regression -**↑** CTL responses	(109)
αPD-1 + α4-1BB	B16-F10 melanoma	-complete regression -**↑** effector/memory differentiation -**↑** CD8/Treg ratio -**↑** effector function -**↑** liver toxicity	(187)
	CT26 colon adenocarcinoma	-Compete tumor rejection -**↑** T cell tumor infiltrate -**↑** T cell effector responses	(186)
psPD-1 + p4-1BBL	H22 hepatocarcinoma	-**↑** anti-tumor immunity-↓ tumor burden -**↑** T cell effector function	(184)
αPD-L1 + α4-1BB	ID-8 ovarian adenocarcinoma	-**↑** survival -**↑** tumor infiltrate-↓ Treg infiltrate -**↑** T cell effector function	(185)
**α*4-1BB *+* Radiotherapy*			
Single dose or fractionated radiation	EMT6 mammary carcinoma M109 lung carcinoma	-↓ tumor burden (high dose)	(196)
Focal radiation + α4-1BB + αCTLA-4	GL261 glioma	-**↑** long-term survival -**↑** T cell brain -CD4^+^ T cell dependent -Established protective immunity	(114)
Whole brain irradiation	GL261 glioma	-**↑** survival -Complete eradication -**↑** TIL	(116)
**α*4-1BB *+* Chemotherapy*			
Cisplatin + α4-1BB	CT26 colon adenocarcinoma	-60% complete regression -↓ lymphopenia -↓ nephrotoxicity	(118)
Cisplatin α4-1BB + αPD-1	ID8 ovarian adenocarcinoma	-**↑** survival -↓ tumor volume -Long lasting immunity	(197)
5-fluorouracil + α4-1BB	Renca renal cell carcinoma	-CD8^+^ T cell mediated -complete eradication -↓ lymphocytopenia -Long-term immunity	(117)
Cyclophosphamide + α4-1BB	B16 melanoma	-CD8^+^ T cell dependent anti-tumor activity -↓ lymphopenia	(198)
Cyclophosphamide + Ad.4-1BB scFv	TC-1 HPV^+^ lung adenocarcinoma	-↓ tumor burden -**↑** survival -↓ Treg infiltrates	(199)
**α*4-1BB *+* Cetuximab*	EGFR^+^ SCC4, SCC6, squamous cell carcinomas EGFR^+^ PC1 pancreatic adenocarcinoma T84 and HCT116 colorectal carcinoma	-**↑** survival-Complete regression -**↑** ADCC -**↑** 4-1BB^+^ NK cells -**↑** NK effector function	(141)
**α*4-1BB *+* Rituximab*	CD20^+^ B cell lymphoma		(142)
**α*4-1BB *+* Trastuzumab*	HER2^+^ breast cancer		(143)

### Agonist antibodies against 4-1BB

By far the most straightforward and most extensively studied approach to targeting 4-1BB relies on the exquisite specificity of targeted antibodies. Melero et al. were the first group to describe the potent anti-tumor properties of agonist 4-1BB antibodies in eliminating murine P815 mastocytoma and Ag104A sarcoma (122). This landmark work opened the field of 4-1BB-targeted immunotherapy. Kim et al., however, demonstrated that α4-1BB antibodies were ineffective as a monotherapy against subcutaneously implanted B16/D5 melanoma, MCA205 sarcoma, or GL261 glioblastoma when administered systemically over a range of doses (123). Interestingly though, systemic monotherapy was effective against intracranially implanted MCA205 and GL261 tumors, suggesting that efficacy of agonist therapy relies heavily on microenvironmental factors as well as intrinsic tumor-resistance mechanisms. In a model of plasmacytoma, May demonstrated that a critical effect of α4-1BB-mediated tumor regression lies in the ability of CD8 T cells from treated mice to survive and avoid AICD (124). Moreover, α4-1BB antibody therapy is dependent on IFNγ, as CD8 T cells were incapable of trafficking to the tumor site in IFNγ-deficient mice (125). The use of 4-1BB antibodies further provides unique advantages over other 4-1BB targeted modalities. For instance, binding of the Fc region of the 4-1BB antibody to Fc receptors may activate NK cells. Moreover, these NK cells then express 4-1BB and in so doing, become targets for immunotherapy (126). Additionally, Martinez-Forero et al. demonstrated the mechanism of α4-1BB antibody binding and internalization into endosomal compartments and subsequent K63 polyubiquitination necessary to recruit TRAF2 and initiate the 4-1BB signaling cascade (127). Importantly, Galectin-9 contributes to the stabilization of 4-1BB for multimerization and ligand binding and stimulation, demonstrating a role for cell surface glycoproteins in mediating receptor signaling (95). While the strong anti-tumor immunity promoted by 4-1BB agonist antibodies can engender serious immune-mediated pathology not evident through some other therapeutic modalities (91, 94, 128), the use of agonist antibodies in combination with other cancer therapeutics may help alleviate these detrimental side effects.

### Soluble 4-1BBL

A surrogate approach to 4-1BB-targeted antibodies lies in the use of the natural 4-1BB ligand to stimulate anti-tumor T cell responses. Mouse forestomach cancer cells transfected with DNA encoding 4-1BBL were capable of doubling the cytotoxicity of tumor infiltrating T cells over untransfected cells, demonstrating the potential of 4-1BBL expression as an alternate means to target 4-1BB therapeutically (129). Transduction of 4-1BBL into other tumor cell lines has also shown therapeutic potential (130–132), particularly the transduction of 4-1BBL into K562 leukemic cells to expand both T cells and NK cells (133, 134). Furthermore, work from the Shirwan lab has elegantly demonstrated the therapeutic effect of a streptavidinated 4-1BBL (SA-4-1BBL) complex to induce effective anti-tumor immune responses. Firstly, subcutaneous administration of SA-4-1BBL potently stimulated both CD8 and CD4 T cell proliferation compared to equal doses of a 4-1BB agonist antibody (94) without a dramatic increase in inflammatory cytokines exhibited by antibody administration. Further, SA-4-1BBL induced less lymphadenopathy and splenomegaly than antibody therapy, suggesting that SA-4-1BBL has a higher therapeutic index. In this study, however, the therapeutic index of systemic administration of SA-4-1BBL was never tested. Moreover, SA-4-1BBL evoked strong anti-tumor effects when given as a vaccine adjuvant in the murine TC-1 HPV-driven tumor model (135–137), or in a Survivin^+^ lung carcinoma model (136, 138). This potential role of 4-1BB agonists as vaccine adjuvants suggests potential future combinations with other TNF (e.g., CD40) or innate (e.g., TLR9) agonists in this setting. The pre-clinical data with SA-4-1BBL suggest that if a clinically suitable multimeric form of 4-1BBL can be developed; it may offer a compelling alternative to 4-1BB agonist antibody mediated stimulation for tumor immunotherapy.

### Aptamers

An alternate means of targeting 4-1BB entails the use of oligonucleotide aptamer technology, pioneered in the Gilboa lab (139). Aptamers are single-stranded oligonucleotides designed through an enrichment process to develop a unique structure capable of binding a given target protein, usually for therapeutic purposes. As 4-1BB most efficiently signals through a multimer complex, so too do 4-1BB bivalent aptamer conjugates more efficiently co-stimulate CD8 T cells. Bivalent aptamers co-stimulated CD8 T cell proliferation with similar potency to agonist antibodies when injected systemically, and elicited approximately two-fold more IFNγ production. These 4-1BB aptamers were thus able to protect mice from P815 mastocytoma tumors with comparable efficacy to antibody monotherapy (119). Much like antibodies, aptamers can be conjugated to various cargos in order to enhance therapeutic benefit (120). Berezhnoy et al. demonstrated that the addition of a siRNA targeting the mTOR pathway conjugated to a 4-1BB aptamer was able to efficiently inhibit Raptor to suppress mTORC1 activity and enhance the persistence of T cells exhibiting a memory phenotype. Moreover, T cells more effectively controlled growth of murine B16 melanomas when mice were administered 4-1BB/Raptor compared to 4-1BB aptamer or rapamycin monotherapies (121). Aptamers can also be conjugated to targeting motifs allowing for the trafficking and close juxtaposition of effector T cells with tumor tissue. Conjugation of a 4-1BB aptamer to a second aptamer targeting the prostate-specific membrane antigen (PSMA) inhibited growth of PSMA expressing tumors and prolonged survival in half of mice receiving the 4-1BB/PSMA aptamer conjugate (140). In a similar vein, 4-1BB/VEGF aptamer conjugates were able to enhance T cell proliferation when administered systemically to mice bearing subcutaneous 4T1 mammary carcinomas or MCA induced fibrosarcoma in a VEGF-dependent manner (128). Notably, the 4-1BB aptamer, 4-1BB/PSMA, and 4-1BB/VEGF conjugates did not induce significant pathology associated with systemic administration of 4-1BB antibodies, as CD8 T cell infiltratration into the spleens and livers was markedly reduced in aptamer-treated mice. Neither the pharmacodynamics of α4-1BB aptamers, nor how the 4-1BB aptamer/receptor interaction mediates signaling into the T cell has been extensively studied. Whether this interaction disrupts 4-1BB/4-1BBL interactions, and by what mechanism the aptamer lessens liver inflammation relative to 4-1BB antibody therapy remains to be determined. Further, 4-1BB targeted antibodies have the added benefit of inducing antibody-dependent cell-mediated cytotoxicity (ADCC) through Fc/Fc receptor interactions on NK cells as a dual arm of therapeutic efficacy (126, 141–143), which would be lacking in aptamer conjugates. Oligonucleotide aptamers, on the other hand, may act as stimuli for nucleotide sensing pathways in innate immune cells and thereby promote activation of antigen-presenting cells. Regardless, the enhanced therapeutic index of 4-1BB targeted aptamers supports their potential translation into the clinic.

## Effects of 4-1BB Agonists on Tumor Progression

Regardless of modality, 4-1BB-targeted therapies potently modulate anti-tumor immune responses to effectively treat a variety of cancers. Tumor cells expressing 4-1BB scFv potently stimulate anti-tumor effector responses (144, 145). In addition, 4-1BB targeted immunotherapy has demonstrated great potential in treating floor of mouth squamous cell cancer (146), lymphoma (96), hepatocellular carcinoma (147, 148), and colon cancer to name a few (149, 150).

The anti-tumor potential of α4-1BB therapy stems from the ability to modulate the tumor microenvironment, largely by promoting a type 1 cytokine response (151). Palazon et al. demonstrated that 4-1BB is up-regulated in limited oxygen environments (152), particularly in hypoxic tumor environments potentially enhancing the selectivity of 4-1BB agonists for cells in the tumor. Ye also established that 4-1BB can act as a marker for tumor-reactive T cells (153). Work of Ju et al. showed that 4-1BB agonist antibodies enhance anti-tumor responses by inducing a CD8^+^CD11c^+^ T cell population with enhanced IFNγ activity (56, 154). Perhaps most strikingly, α4-1BB therapy is capable of inducing a potently cytotoxic T cell phenotype mediated by the T-box transcription factor Eomesodermin (61, 155), which is required for 4-1BB-mediated tumor therapy (156). Further, perforin and granzyme act together during α4-1BB therapy to eradicate established murine lymphomas (157), adding to the body of work demonstrating the role of 4-1BB in enhancing cytotoxic responses. While 4-1BB predominantly acts on T cells, depletion of dendritic cells impairs the anti-tumor effects of α4-1BB, suggesting a role for DCs as well in anti-tumor 4-1BB agonist immunotherapy (158). Moreover, α4-1BB therapy can act on 4-1BB^+^ endothelial cells to increase T cell recruitment into tumor sites and sites of inflammation (159).

## 4-1BB Agonist Antibodies in the Clinic: Adverse Events and the Potential to Overcome Them

Expression of 4-1BB correlates well with effective anti-tumor immune responses (153); however, 4-1BB agonist antibodies can induce a variety of pathologies that may limit their utility in patients.

In the setting of natural immunity, 4-1BB signaling has been implicated in mediating the pathogenesis of herpetic stromal keratitis (an HSV-1 associated eye infection that can lead to glaucoma and/or corneal scarring) (160), atherosclerosis (161, 162), obesity-induced inflammation (163–165), allograft rejection (166–169), lung inflammation, and airway hyper-responsiveness (27), as well as infection-induced fetal rejection during pregnancy (170). Though, mild and manageable, the potential for 4-1BB to precipitate auto-reactive pathologies should be considered in planning the management of 4-1BB agonist treated patients.

The most clinically relevant adverse events associated with the use of 4-1BB agonist antibodies involve defects in immune homeostasis (e.g., neutropenia, thrombocytopenia, and reduced B cell numbers) (171, 172), as well as moderate to severe liver inflammation characterized by immune infiltration and concomitant elevation in serum levels of liver transaminases (e.g., AST, ALT). Dubrot et al. first demonstrated a polyclonal influx of CD8 T cells into the livers of mice bearing MC38 colon carcinomas which correlated with an increase in transaminase levels (173). Wang et al. further demonstrated in a mouse model of chronic Hepatitis C infection that, 4-1BB stimulation promotes hepatic fibrosis and progression to hepatocellular carcinoma (174). This progression was mediated by production of IFNγ from CD8 infiltrates which drove CD11b^+^ macrophages to increase production of inflammatory cytokines. Interestingly, Lin et al. established a role for the glucocorticoid-induced TNF-related receptor (GITR) in mediating 4-1BB-induced liver pathology (175). GITR^−/−^ mice treated with 4-1BB antibodies demonstrated reduced splenomegaly as well as decreases in both ALT and AST levels associated with liver damage. GITR knockout mice also had fewer T cell infiltrates into the liver as well as fewer 4-1BB^+^ regulatory T cells and dendritic cells in the spleens and lymph nodes, suggesting that GITR may play a role in systemic 4-1BB expression.

In translating 4-1BB agonists into the setting of clinical oncology, responses to α4-1BB have been impressive, including partial remission and some stable disease; however, adverse events have complicated progression of 4-1BB agonists into late stage clinical trials. As reviewed by Ascierto et al. (91), in a Phase I/II trial conducted by Bristol–Myers Squibb (BMS) using α4-1BB monoclonal antibodies for advanced or metastatic solid tumors (NCT00309023), cases of low grade fatigue were witnessed as well as grade 2+ neutropenia, leukopenia, thrombocytopenia, and increases in AST and ALT. This mild and manageable toxicity profile led to a Phase II study of 4-1BB antibodies for previously treated stage IV melanoma patients (NCT00612664). Unfortunately, though, this study was terminated due to high incidence of severe (Grade IV), and potentially fatal, hepatitis. These severe adverse events led to withdrawal or termination of several other ongoing and approved Phase I trials designed to discover the breadth and potency of the anti-tumor effects of 4-1BB agonist immunotherapy (NCT00803374, NCT00309023, NCT00461110, NCT00351325). There is room for improvement in clinical management of 4-1BB induced hepatitis as steroids alone may not be sufficient to ameliorate severe hepatitis without the addition of myelosuppressive agents. Despite these setbacks, the therapeutic potential of 4-1BB agonist antibodies, particularly in combination with other immune and traditional cancer therapies, has led to a revival of clinical 4-1BB antibody development.

## Potential for Combinatorial Therapies

Though liver toxicity is a major concern in the treatment of advanced cancers, addition of 4-1BB agonists to other therapeutic modalities could potentiate stronger anti-tumor responses while necessitating reduced dosing, thus limiting severity of 4-1BB associated adverse events. Preclinical studies have shown cooperative and even synergistic therapeutic benefit by combining 4-1BB agonists with multiple anti-tumor therapies including IL-12 (176), IFNα (177), vaccination (102, 149, 178–180), as well as various other combinations (99, 101, 141–143). The most alluring combinations, however, are those that combine 4-1BB agonists with therapies that are already approved or in clinical trials, particularly T cell immune checkpoint blockade.

### Anti-4-1BB in combination with gene therapy and oncolytic virotherapy

The use of oncolytic viruses to treat cancer or gene therapy to introduce novel genes into the tumor microenvironment has begun to gain new life in recent years (181). Early gene therapy approaches to tumor vaccination sought to activate 4-1BB as an adjuvant. Incorporating 4-1BB ligand co-stimulation into viral therapies has also proven especially fruitful (92, 93, 97, 98, 100, 103). Work from Melero first demonstrated that Ag104A sarcomas transfected with both 4-1BBL and B7-1, but neither alone, induced potent anti-tumor responses and cured 60% of treated mice (130). Work from Chen et al. further demonstrated the potent therapeutic benefit of combining 4-1BB targeted therapy with interleukin 12, a cytokine that robustly activates NK cells as well as induces Th1 responses. Intratumoral injection of an adenoviral vector containing the p35 and p40 IL-12 subunits in combination with systemic administration of a 4-1BB antibody led to a dose-dependent increase in survival and complete rejection of MCA26 colon adenocarcinomas and non-immunogenic B16-F10 melanomas (104, 106). Intratumoral and systemic administration of 4-1BBL and IL-12 showed similar effects (105, 131, 182), and, in fact, intratumoral 4-1BBL may induce a more robust secondary tumor response than systemic 4-1BB antibodies (131). In a similar vein, while intratumoral implantation of IL-12 transfected DCs showed some anti-tumor effects, addition of systemic 4-1BB agonist antibodies led to complete cures in some cases of both directly injected and untreated contralateral MC38 colon adenocarcinomas (150). These early experiments using viral vectors clearly demonstrated the adjuvant properties of 4-1BB co-stimulation and opened the doors to additional dual therapies involving 4-1BB. Clearly, 4-1BB activation may prove a potent combination strategy as second generation oncolytics emerge which co-express co-stimulatory ligands and/or cytokines.

### Combining 4-1BB agonists with CTLA-4 blockade

The FDA approval of anti-CTLA-4 (Ipilimumab) checkpoint blockade for the treatment of advanced stage melanoma has made this an attractive therapeutic for combination with 4-1BB agonists. While a Phase I trial was approved to determine the therapeutic benefit of combining 4-1BB agonists with Ipilimumab (NCT00803374), this trial was withdrawn prior to opening enrollment due to the liver toxicity observed in the melanoma monotherapy trial. The synergistic therapeutic potential of the α4-1BB/αCTLA-4 dual therapy in preclinical models, however, cannot be overlooked. Three doses of α4-1BB/αCTLA-4 administered to mice bearing B16 melanomas demonstrated a potently synergistic curative effect (183). Combination therapy led to changes in the tumor microenvironment including increases in CD8 T cell infiltration as well as significantly increased CD8/Treg, CD4 Teff/Treg, and CD8/MDSC ratios, which favor tumor clearance and successful treatment. Moreover, dual therapy increased the effector capabilities of T cells in the tumor microenvironment. It should be noted though, that this synergism was only demonstrated in combination with an irradiated tumor vaccine expressing Flt-3 ligand (FVAX). Further evidence of the therapeutic benefit of α4-1BB/αCTLA-4 was demonstrated by the injection of prostate tumors transfected with a plasmid containing 4-1BBL. Enhanced survival benefit and complete tumor rejection were evidenced when combined with systemic αCTLA-4 antibody therapy, leading to long-term immunological protection (109).

The most convincing argument for combining α4-1BB with αCTLA-4 stems from the ability of each therapy to not only potentiate stronger anti-tumor responses, but to also mutually ameliorate the side effects of each monotherapy. Kocak et al. demonstrated that systemic administration of α4-1BB/αCTLA-4 antibody therapy, while ineffective in treating B16 melanoma, was effective in clearing poorly immunogenic MC38 colon adenocarcinoma (110). Most strikingly, whereas αCTLA-4 induced the production of anti-dsDNA antibodies, leading to autoimmune-like syndromes, addition of α4-1BB appeared to alleviate antibody deposition and development of lupus-like pathology in the kidney. Moreover, α4-1BB induced an influx of T cell infiltrates into the liver, leading to hepatitis; however, addition of αCTLA-4 reduced cellular infiltration and liver pathology. The clear therapeutic benefit and alleviation of pathology demonstrated by Kocak suggest that this α4-1BB/αCTLA-4 combination should be prioritized for clinical translation.

### Targeting 4-1BB and the PD-1/PD-L1 axis to elicit potent anti-tumor effects

Another checkpoint receptor with potential therapeutic synergy in combination with α4-1BB is the programmed death-1 (PD-1) pathway. Targeting the PD-1 pathway by blocking PD-1 (108), or by blocking Programed death-ligand 1 (PD-L1) (107), has evoked impressive clinical responses to melanoma, non-small-cell lung cancer, and renal cancer. The recent approval of the PD-1 blocking antibodies Nivolumab and Pembrolizumab for the treatment of melanoma validates the effectiveness of αPD-1 as a monotherapy, confirms the attractiveness of a lower toxicity profile than Ipilimumab, and suggests that αPD-1 may offer a more appealing partner for α4-1BB co-therapy.

In one of the first preclinical models of dual therapies targeting the 4-1BB and PD-1 pathways, Xiao et al. demonstrated that systemic administration of soluble PD-1 to inhibit PD-L1 synergized well with implantation of H22 hepatocarinomas transfected with 4-1BBL by increasing anti-tumor cytotoxicity and decreasing tumor burden compared to either monotherapy (184). In an ovarian cancer model, co-administration of a PD-L1 antagonist with α4-1BB and a cellular vaccine expressing GM-CSF (GVAX) let to increases in both CD4 effector and CD8 T cell infiltrates into the tumor with a concomitant decrease in regulatory T cells (185). Much like with α4-1BB/αCTLA-4 therapy, this combination increased IFNγ and TNFα production in the tumor microenvironment. PD-1 blocking antibodies in combination with 4-1BB agonists have also shown increased therapeutic potential toward subcutaneously implanted CT26 colon carcinoma (186) or B16/F10 melanoma (187). Interestingly, Chen et al. showed that PD-1 blockade increased 4-1BB expression on CD8 T cells and α4-1BB conversely induced PD-1 expression, thus pointing toward a mechanism of potential synergy in the combination setting. Furthermore, in this study, dual therapy increased the CD8/Treg ratio in the tumor as well as the potent effector capacity of T cells. Unfortunately, though, co-administration of α4-1BB and αPD-1 appeared to exacerbate α4-1BB associated toxicities at moderate to high doses of 4-1BB agonist treatment in mice. At both 1 and 5 mg/kg doses of α4-1BB, ALT and AST levels were increased almost two-fold over α4-1BB alone. Moreover, α4-1BB/αPD-1 therapy failed to ameliorate, and even worsened, α4-1BB mediated thrombocytopenia, lymphopenia, and neutropenia at the higher doses. At the lowest dose of α4-1BB, however, no increased toxicity was observed for the α4-1BB/αPD-1 combination relative to the same dose of α4-1BB alone. These results strongly suggest caution in choosing combination therapies, and that trial design should include conservative dosing in initial cohorts with the potential for escalation after demonstration of safety.

### Combining TNF receptor agonists: Potential for 4-1BB and OX-40 dual therapy

Another avenue for combination therapy that may have therapeutic potential engages combination of α4-1BB with agonists of other co-stimulatory TNFR family members. Clinical trials targeting the OX-40 pathway for tumor therapy are currently underway (NCT01862900, NCT02274155, NCT01303705). Moreover, the co-stimulatory nature of both 4-1BB and OX-40 receptors as well as diversity in their expression patterns may offer potential synergism between these two therapies in the clinic (188, 189). Work from Lee et al. demonstrated that activating both OX-40 and 4-1BB enhanced CD8 T cell proliferation, survival, and effector function over either monotherapy in response to staphylococcal enterotoxin A (SEA) stimulation (190). In the context of a prime/boost vaccine using a recombinant vaccinia virus (VV) vector encoding the OVA-peptide, α4-1BB alone enhanced CD8 T cell expansion and memory formation, whereas the α4-1BB/αOX-40 combination was able to expand both CD4 and CD8 responses (191). Further, OX-40 and 4-1BB co-stimulation may advantageously regulate Treg function as well (192). For instance, work from St. Rose et al. suggests that enhanced IFNγ production during dual co-stimulation regulates expression of the IL-2 receptor (CD25), limiting the expansion of regulatory T cells (193). Contrarily though, one report demonstrated enhanced expansion of Tregs during 4-1BB stimulation (194), while another showed that α4-1BB inhibits the suppressive capacity of CD4^+^CD25^+^ regulatory T cells (195), and yet a third study reported suppressed conversion of CD4^+^ T effector cells into Tregs during 4-1BB stimulation (111). Thus, the functional consequences of 4-1BB activation upon Tregs remains contentious, and further studies are necessary, particularly to clarify the impact of α4-1BB on human Treg expansion and suppressive capacity.

Significant to the field of tumor immunology, Bandyopadhyay et al. showed that dual co-stimulation through α4-1BB/αOX-40 fueled T cell expansion but not effector function in a murine model of responses toward tolerized self-antigens (113). This study, while suggestive of implications on tumor immunotherapy, did not take into account the vast array of neoantigens or polyclonal nature of the adaptive immune response in a natural setting. Further, the therapeutic potential of combining dual co-stimulation with whole tumor vaccines compared to peptide vaccination was clearly demonstrated by Cuadros et al. This strategy produced stronger T cell responses compared to peptide vaccination and fostered complete tumor rejection when combined with α4-1BB/αOX-40 therapy, further demonstrating the need to generate an oligoclonal anti-tumor response to efficiently eliminate certain tumors (112). The ability to boost both CD8 and CD4 T cell responses while, at the same time, suppressing Treg expansion will likely make 4-1BB and OX-40 agonists attractive combination partners in the clinic.

### Coupling 4-1BB agonists with radiation therapy

Front-line radiation therapy is the current standard of care for many malignancies, often eliciting objective responses. More recently, the capacity of radiotherapy to awaken and augment dormant tumor immune responses has been demonstrated both in pre-clinical studies and clinical trials. Shi et al. demonstrated that high doses of radiation can induce expression of 4-1BBL on some murine tumors (196). In addition, a single dose or multiple, fractionated doses of radiation given prior to systemic administration of 4-1BB agonists induced partial tumor regression in murine models of lung and breast cancer. In particular, cancers of the brain and nervous system may benefit from the potential of α4-1BB/radiotherapy combinations. As neurological cancers are highly radiosensitive and high doses or repeated exposure lead to cognitive impairment, therapies that permit lower dosages with increased anti-tumor effects are sorely needed. In one of the first studies seeking to combine 4-1BB antibodies with radiation, mice implanted with intracranial GL261 gliomas were treated with whole brain irradiation in combination with systemic α4-1BB. This combination therapy dramatically increased survival, reduced total tumor volume, and increased lymphocyte infiltration with the acquisition of durable systemic immunological memory (116). Belcaid et al. further demonstrated that while αCTLA-4 therapy in combination with focal radiation increased the overall survival of mice intracranially implanted with GL261, radiotherapy in combination with αCTLA-4/α4-1BB further increased survival and led to long-term immunological protection (114). Interestingly, investigators found that CD4 T cells played a critical role in mediating this effect, as CD4 but not CD8 T cell depletion abrogated the therapeutic response. The capacity of 4-1BB agonist antibody to expand and empower tumor-specific T cell responses unleashed following radiotherapy clearly will make this a desirable and accessible therapy in the clinic for both corporeal and, potentially, CNS malignancies.

### Advantageous combinations of chemotherapy and 4-1BB activation

While various chemotherapeutic drugs have demonstrated anti-tumor responses and become the standard of care for both hematological and solid tissue malignancies, most tumors become refractive to therapy, demonstrating a need for combination therapies to overcome resistance. As chemotherapy regimens can induce T cell co-stimulatory receptors, in particular 4-1BB (115), and elicit tumor antigen release, immunotherapy has emerged as a key candidate for combination with chemotherapy.

Agonist 4-1BB therapy with chemotherapy has proven effective pre-clinically in multiple murine tumor models. Ju et al. demonstrated that either α4-1BB or 5-fluorouracil (5-FU) alone did little to treat RCC; however, the combination of α4-1BB with 5-FU led to profound tumor regressions and increased overall survival rates in dual treated mice (117). Further, adding α4-1BB with the DNA-alkylating platinum-containing derivatives, particularly cisplatin, produced cooperative anti-tumor responses and increased survival (118). Not only did α4-1BB/cisplatin induce complete rejection of CT26 colon adenocarcinoma, but addition of 4-1BB agonists also afforded protection from cisplatin-induced nephrotoxicity. In addition, cisplatin in combination with PD-1 blockade and α4-1BB antibody therapy improved responses in a murine model of ovarian cancer, increasing the overall survival rate in a CD8 T cell-dependent fashion (197). Moreover, α4-1BB acts cooperatively with cyclophosphamide (CTX) therapy. While CTX, given early, showed moderate increases in median survival, CTX in combination with α4-1BB increased overall survival by eliciting polyclonal expansion of anti-tumor T cells with significantly enhanced effector function (198). In a similar fashion, CTX treatment followed by intratumoral injection of an adenoviral vector encoding an agonistic scFv targeting 4-1BB synergized to treat murine TC-1 lung adenocarcinomas, boosting T cell proliferation while suppressing Treg expansion (199). Clinical application of 4-1BB agonist and chemotherapy combinations will require careful design to avoid bystander killing of the 4-1BB amplified T cells by the cytotoxic agent; however, the preclincal data suggest translational promise for 4-1BB to augment the effect of selected chemotherapies.

### Potentiation of adoptive T cell therapy by 4-1BB activation

*Ex vivo* expansion and re-infusion of a patient’s own tumor-specific T cells, known as adoptive cell therapy (ACT), has become a potent new class of immunotherapy, particularly for melanoma. ACT seeks to either expand a patient’s own endogenous anti-tumor T cells, or alternatively, to genetically engineer endogenous T cells with chimeric antigen receptors (CARs) in order to redirect them to the tumor. While CARs offer exceptional anti-tumor specificity and effector function, adoptive transfer of a patient’s own tumor reactive TIL or PMBC initiates immunity against a broader range of tumor-associated antigens, thereby reducing the chance of tumor immune escape through antigen loss. Only recently has the role of 4-1BB in demarking tumor reactive T cells, and in rapidly and robustly expanding T cells for ACT, been appreciated and instituted into TIL expansion protocols (153).

Separate work from Strome et al. and Li et al. demonstrated the synergy of 4-1BB agonists used in combination with adoptively transferred T cells to treat murine lung metastases (200, 201). Moreover, in a hallmark paper, Maus et al. showed that the capacity of K562 cells used as artificial antigen presenting cells (aAPC) to expand patient TIL was dramatically enhanced by co-expression of 4-1BBL (133). This model has now become the standard protocol for *ex vivo* expansion of T cells for adoptive transfer. Work from Chacon et al. further uncovered the potential of adding 4-1BB agonist antibody stimulation after expansion of TIL in human melanoma, particularly in preventing AICD of TIL *ex vivo* (202). In order to gain enough T cells from patient TIL samples for ACT, TIL samples undergo a rapid expansion protocol (REP). By adding α4-1BB post-REP, Chacon demonstrated increased polyclonal expansion of CD8^+^ TIL. These cells were highly functional and capable of responding to antigenic restimulation. Choi et al. showed in similar fashion that tumor-antigen-specific T cells can be harvested and expanded from a patient’s peripheral blood much more rapidly than traditional TIL expansion protocols permit via the addition of 4-1BB agonists (203). Care should be taken, however, in using α4-1BB to expand patient lymphocytes prior to reinfusion, as, despite preferential expansion of CD8^+^ T cells, 4-1BB agonists may also augment other 4-1BB-expressing cell types including DCs, NK cells, and Tregs. Goldstein et al. showed, for example, that 4-1BB also is present on a population of Treg cells capable of suppressing anti-tumor effector function (204).

Engineering CAR T cells serves as an alternate means of adoptive T cell transfer in which patient T cells are transgenically altered *ex vivo* to express a tumor-targeted antibody Fab linked to the TCR signaling machinery. Early generation CARs bearing tumor-targeted scFV with CD3ζ demonstrated some anti-tumor potential, but failed to persist long term. Adding co-stimulatory endodomains (via CD28 and/or 4-1BB), however, greatly increased tumor-killing potential and *in vivo* persistence of adoptively transferred CAR T cells (205). These αCD19-BB-ζ CARs prove to be highly cytotoxic cells capable of potentiating strong anti-leukemia activity (206). Further, in a small pilot study, αCD20 CARs engineered with 4-1BB endodomains produced therapeutic benefit against relapsed indolent B-cell and mantle cell lymphomas (207). From this and many other CAR T cell trials, it appears clear that the presence of the 4-1BB signaling domain affords advantages in both persistence and effector function to adoptively transferred CAR T cells whether alone or in combination with the CD28 endodomain.

Manipulating 4-1BB in the adoptive transfer setting to treat cancer is an expanding area of interest within the field of immunotherapy. A multitude of upcoming CAR T cell trials in both hematologic and solid tumors will test the value of the 4-1BB endodomain in enhancing their anti-tumor activity and *in vivo* persistence. Also, the potency of 4-1BB agonist antibodies in selecting, expanding, and conditioning the most effective tumor-specific CD8^+^ T cells for ACT will also be thoroughly tested in upcoming studies.

### Other therapies employing 4-1BB

Many other therapeutic modalities have incorporated 4-1BB agonists to enhance weak anti-tumor responses. Work from Kohrt et al. elegantly demonstrated the therapeutic potential of combining α4-1BB antibodies with approved tumor-targeted antibody therapies. In one study, combining α4-1BB with αCD20 antibodies profoundly enhanced NK cell-mediated anti-lymphoma activity. By combining sequential injections of αCD20 before administration of α4-1BB, Kohrt demonstrated that αCD20 administration enhanced NK cell tumor-killing capacity through antibody-dependent cell-mediated cytotoxicity (ADCC). This led to increases in anti-tumor 4-1BB^+^ NK cells that then served as targets for α4-1BB therapy (142). Kohrt went further, demonstrating that NK cells from patients with various B cell lymphomas and leukemias upregulated 4-1BB during culture with Rituximab-coated tumor cells, offering a proof of principle that Rituximab and α4-1BB work well as a dual therapy to treat B cell malignancies. In a similar fashion, treatment with trastuzumab or cetuximab prior to α4-1BB agonist therapy boosted ADCC and NK cell responses to HER2^+^ breast cancer or EGFR^+^ head and neck and colorectal carcinomas, respectively (143). These pre-clinical findings have fostered clinical trials of α4-1BB antibodies in combination with these tumor-specific antibodies.

## 4-1BB-Targeted Clinical Trials in Progress

Impressive preclinical anti-tumor potential (Table 1) has progressed 4-1BB targeted therapies into clinical trials. Pfizer is currently recruiting patients for a Phase I trial to determine the safety profile and potential of the combination of α4-1BB (PF-05082566) with Pembrolizumab for the treatment of advanced solid tumors (NCT02179918). Additionally, patients are being recruited for a Phase I trial of α4-1BB (PF-05082566) in conjunction with Rituximab (αCD20) for the treatment of Non-Hodgkin’s lymphoma (NCT01307267).

Further, BMS is currently recruiting for multiple trials including a Phase I safety trial of Urelumab (BMS-663513) for advanced stage metastatic solid tumors and relapsed/refractory B cell Non-Hodgkin’s lymphoma (NCT01471210). Recruitment is also ongoing for a trial combining Elotuzumab (targeting SLAMF7) in combination with Lirilumab (targeting KIR receptors on NK cells) or Urelumab (α4-1BB) for the treatment of multiple myeloma (NCT02252263). BMS has also opened Phase I trials combining Urelumab with Cetuximab for metastatic colorectal cancer and head and neck cancer (NCT02110082), combining Urelumab with Rituximab for Non-Hodgkin’s lymphoma (NCT01775631), and Urelumab with Nivolumab (αPD-1) for the treatment of solid tumors and Non-Hodkin’s lymphoma (NCT02253992). All of these trials are still Phase I, and results are eagerly awaited.

The majority of these trials focus on modulating NK cell responses in order to not only boost ADCC, but to also activate and expand anti-tumor NK cells. While both the BMS and Pfizer/Merck trials of α4-1BB/αPD-1 combination therapy seek to translate the impressive pre-clinical efficacy of this combination, it remains to be seen, especially for Urelumab, whether patients will be able to tolerate sufficient doses of these two antibodies to realize this potential. We believe the greatest clinical impact of 4-1BB agonist antibodies will come in future combination trials with CTLA-4 blockade in which both therapeutic synergy and reduction in one another’s immune related adverse events appears likely. This would be a unique case in which both agents, which are currently being under-dosed in the clinic to minimize side effects, could be administered in higher doses than as monotherapies and with a better safety profile. Tumor selective 4-1BB antibodies are also in development using, for example, the Cytomix probody technology, which would allow maximum efficacious activation of 4-1BB alone and in combinations without fear of high grade liver inflammation (208). These developments which separate the anti-tumor from the liver inflammatory effects of 4-1BB antibodies will dramatically accelerate the progress of this extraordinarily promising immunotherapy toward FDA approval.

## Future Directions

Activation of the 4-1BB receptor has proven exquisitely effective in treating a wide range of murine tumors. Hepatic toxicities elicited by α4-1BB therapy, however, have limited its progression into Phase II/III clinical trials (91). Fortunately, new therapeutic modalities using 4-1BB targeted aptamers (119, 128, 140), as well as therapeutic combinations with other immuno-modulatory and traditional anti-cancer treatments, have renewed excitement for the use of 4-1BB agonists in the clinic. The most significant dual therapies may lie in combinations of α4-1BB with T cell immune checkpoint blockade, in particular αCTLA-4. This combination has demonstrated therapeutic synergy in multiple tumor models (109, 114, 183), and, remarkably, diminishes immune related toxicities compared to each antibody given alone (110). When selecting combination partners for 4-1BB agonist antibodies, both combinatorial efficacy and toxicity should be considered. For example, both αCTLA-4 and αPD-1 synergize with α4-1BB therapeutically; however, αCTLA-4 reduces α4-1BB mediated liver inflammation while αPD-1 seems to exacerbate it (110, 187). Additionally, unlike checkpoint blocking antibodies which restore T cell proliferation and functionality but do little to alter their phenotype, 4-1BB agonists strongly suppress Th17 T cell responses and favor a highly cytotoxic, tumoricidal ThEO T cell phenotype (61, 76). For tumors such as colon cancer in which Th17 polarized T cells play important roles in tumor formation and support, combinations involving α4-1BB which can alter the balance of T cell phenotypes away from a Th17 and toward a Th1 or ThEO polarity could offer uniquely effective therapy. Ongoing efforts to design tumor-selective 4-1BB agonists coupled with pre-clinical studies focused on revealing the detailed cellular and molecular mechanisms by which 4-1BB agonists enhance tumor immunity, alone and in combination with other therapies, predict a significant role for these agents in the future of clinical tumor immunotherapy.

## Conclusion

4-1BB is a co-stimulatory receptor expressed on a variety of cells of the immune system, particularly on CD8^+^ T cells. This broad range of expression, coupled with the ability of 4-1BB to potentiate strong and durable immune effector responses, has made 4-1BB a clinically viable target for cancer immunotherapy. Although 4-1BB can be targeted through a variety of mechanisms, its capacity to treat advanced tumors as a monotherapy is limited. The unique and often synergistic advantages of 4-1BB activation in combination with other therapies, however, suggest a prominent role for these agents in the treatment of multiple types of cancer. Despite early setbacks, 4-1BB agonists may provide a critical piece in assembling combination therapies capable of achieving durable complete responses against advanced cancers.

## Conflict of Interest Statement

The authors declare that the research was conducted in the absence of any commercial or financial relationships that could be construed as a potential conflict of interest.
